# Complete Chloroplast Genome of *Pinus massoniana* (Pinaceae): Gene Rearrangements, Loss of *ndh* Genes, and Short Inverted Repeats Contraction, Expansion

**DOI:** 10.3390/molecules22091528

**Published:** 2017-09-11

**Authors:** ZhouXian Ni, YouJu Ye, Tiandao Bai, Meng Xu, Li-An Xu

**Affiliations:** 1 Co-Innovation Center for Sustainable Forestry in Southern China, Nanjing Forestry University, Nanjing 210037, China; nzhx0627@163.com (Z.N.); yeyj9403@163.com (Y.Y.); btdman20@163.com (T.B.); mengxu412@126.com (M.X.); 2 Forestry College, Guangxi University, Nanning 530004, China

**Keywords:** conifer species, genome annotation, structural inversion, comparative genomics, phylogenetic analysis

## Abstract

The chloroplast genome (CPG) of *Pinus massoniana* belonging to the genus *Pinus* (Pinaceae), which is a primary source of turpentine, was sequenced and analyzed in terms of gene rearrangements, *ndh* genes loss, and the contraction and expansion of short inverted repeats (IRs). *P. massoniana* CPG has a typical quadripartite structure that includes large single copy (LSC) (65,563 bp), small single copy (SSC) (53,230 bp) and two IRs (IRa and IRb, 485 bp). The 108 unique genes were identified, including 73 protein-coding genes, 31 tRNAs, and 4 rRNAs. Most of the 81 simple sequence repeats (SSRs) identified in CPG were mononucleotides motifs of A/T types and located in non-coding regions. Comparisons with related species revealed an inversion (21,556 bp) in the LSC region; *P. massoniana* CPG lacks all 11 intact *ndh* genes (four *ndh* genes lost completely; the five remained truncated as pseudogenes; and the other two *ndh* genes remain as pseudogenes because of short insertions or deletions). A pair of short IRs was found instead of large IRs, and size variations among pine species were observed, which resulted from short insertions or deletions and non-synchronized variations between “IRa” and “IRb”. The results of phylogenetic analyses based on whole CPG sequences of 16 conifers indicated that the whole CPG sequences could be used as a powerful tool in phylogenetic analyses.

## 1. Introduction

The chloroplast genome (CPG) has multiple copies in a chloroplastid, which consists of 110–210 kb of circular DNA [1,2,3]. It has a quadripartite structure containing large and small single copy (LSC and SSC) regions, and two inverted repeats (IRs). There are 110–130 genes in most land plant CPGs [3,4]. With the development of next-generation sequencing, more than 1,000 CPGs have been reported in NCBI (http://www.ncbi.nlm.nih.gov/genomes/GenomesGroup.cgi?taxid=2759&opt=plastid).

Large IRs are typical structures in CPGs, and have sizes ranging from 15 kb to 30 kb [3,5]. Some tRNA and rRNA genes (e.g., *trnaI*-GAU, *trnV*-GAC and 16S rRNA) are located in the large IRs. However, there are some differences in the gene numbers among species because of the contraction or expansion of IRs [5]. In addition, large IRs play important roles in stabilizing the CPG structure [6,7] due to the low rate of nucleotide substitution and enhanced copy-correction activity [5,8]. Thus, the loss of large IRs could result in the shortening of intergenic spaces [6], gene loss, and structural variations in CPGs [7,9]. The loss of large IRs has been confirmed in species in Pinaceae, Taxodiaceae [10,11], Cephalotaxaceae [12] and Legumes [8].

The contraction or expansion of large IRs could cause gene loss in some species’ CPGs, as mentioned above. In addition, gene transfer among chloroplast, mitochondrial, and nuclear genomes could lead to the transfer of some CPG genes to the nuclear genome [13]. A copy of the *accD* gene, which has been lost in the CPGs of *Sciadopitys verticillata*, has been found in the nuclear genome [10]. The *infA* gene has also transferred from the CPGs to the nuclear genome [14]. Additionally, the CPGs of species in Orchidaceae [15], Geraniaceae [16], and Pinaceae [17] lack all of the 11 intact *ndh* genes, which can also be observed in many land plants [15], and is related to gene transfer or *ndh* gene functions [15,17,18]. Besides those mentioned previously, the coding regions of matK, rbcL, and rpoB genes and non-coding regions of atpF-atpH, trnH-psbA, and psbK-psbI have been widely used in phylogenetic analyses of plants [19,20]. Additionally next-generation sequencing allows the LSC, SSC, and IRs regions, and shared protein-coding genes (PCGs), to be used as powerful tools in phylogenetic analyses [5,21].

Pinaceae is the largest family in gymnosperms, which includes three subfamilies, 10 genera and more than 230 species. Most of the species in Pinaceae are forest and timber species distributed in the northern hemisphere. *Pinus* is the only genus in the subfamily Pinoideae, which consists of more than 80 species. The species in *Pinus* are also the main wood and turpentine producers worldwide. In southern China, *Pinus massoniana* is an outstanding wood resource due to its economic value and broad geographic distribution. Additionally, the chloroplast genome plays an important role in studies of phylogenetic analysis, parental analysis, genetic structure, and germplasm resources evaluation, because of its characteristics of uniparental inheritance and conserved sequence.

In the present study, we obtained the complete CPG sequence of *Pinus massoniana*, and describe its gene content and microsatellite distribution. Comparisons with related species for gene content, gene rearrangements, *ndh* gene loss, and the contraction and expansion of short IRs were also performed. A phylogenetic analysis was performed on the basis of the whole CPGs of 16 conifers.

## 2. Results and Discussion

### 2.1. Genome Organization and Comparison with Other Species

**Figure 1:**
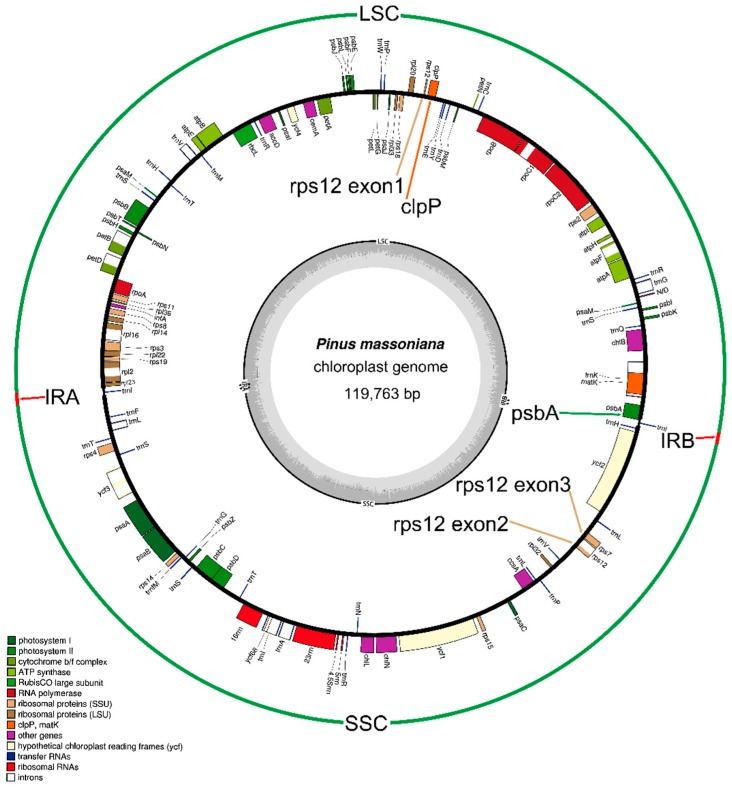
Chloroplast genome annotation map for *Pinus massoniana*. Genes lying outside the circle are transcribed in a clockwise direction, whereas genes inside are transcribed in a counterclockwise direction. Different colors represent different functional groups. The dashed darker and lighter gray in the inner circle denote GC and AT contents of chloroplast genome, respectively. LSC, SSC and IRs means long single copy, small sngle copy, and inverted repeat regions, respectively.

Using 35 primer pairs [22], we obtained the whole CPG sequence (119,763 bp) of *P. massoniana* (GenBank accession number: MF564195), which possesses a typical quadripartite structure including the LSC (65,563 bp) and SSC (53,230 bp) regions and the IRa and IRb pair (485 bp) (Figure 1). Compared with species in Magnoliaceae (159–161 kb), Salicaceae (Angiosperms) (156–171 kb) and Ginkgoaceae (Gymnosperms) (~157 kb), all of the species in *Pinus* have shorter CPGs (116–122 kb) (Supplementary Table S1) [3,23,24]. In addition, we observed differences in CPG sizes among species (*Pinus*) as follows: the CPGs of species in section Pinus (119–122 kb) > CPGs of species in section Parrya and section Cembra (116–118 kb) [25,26]; and the CPGs of species originating from the Americas (>120 kb) > CPGs of species originating from the Eurasian continent (119–120 kb) [22,26,27]. Compared with species in Magnoliaceae, Salicaceae, and Ginkgoaceae (SSC: 18–23 kb and IR: 17–28 kb), the CPGs of species in *Pinus* have longer SSCs (51–55 kb) and shorter IRs (400–500 bp). The previous studies revealed that the contraction of IRs could result in the expansion of SSC regions in *Pinus* [5,21].

The CPG of *P. massoniana* has a similar GC content to those of other *Pinus* species. However, few differences in the GC contents were found among the CPGs. The GC content is highest in the SSC region (39.4%), moderate in the LSC region (37.9%), and lowest in the IR region (36.3%). The base contents are different from *Oryza minuta* CPGs, in which the GC content is unequally distributed in different regions, and the IR regions have the highest GC percentage [1].

### 2.2. Gene Contents

We identified 113 genes in the CPG of Masson pine, of which 108 are unique genes; these consist of 73 PCGs, 31 tRNA genes, and 4 rRNA genes (Table 1, Figure 1). The gene contents are similar to those of *Pinus taiwanensis*, but there are two additional genes (*ycf12* and *ycf68*, unknown functions) than in *Pinus bungeana* [25,28]. In addition to the two PCGs (*rps12* and *ycf3*) that have two introns each, six PCGs (*atpF*, *petB*, *petD*, *rpl2*, *rpl16*, and *rpoC1*) and six tRNA genes (*trnA-UGC*, *trnG-GCC*, *trnI-GAU*, *trnK-UUU*, *trnL-UAA* and *trnV-UAC*) contain single introns. Like the clade known as the IR-lacking clade (IRLC) of legumes, *clpP* intron loss was also observed in *P. massoniana* CPGs, although the *clpP* gene has two introns in several angiosperms and *G. biloba* [3,29]. The *clpP* intron loss was also observed in the *Passiflora edulis* CPGs [4,29]. *trnK-UUU* has the longest intron (2501 bp), in which the *matK* gene is located according to a previous report on Gentiana [30].

The LSC of the *P. massoniana* CPG contains 73 genes, including 17 tRNA genes and 56 PCGs. Additionally, 17 tRNA genes, 18 PCGs, and 4 rRNA genes are located in the SSC, while IRs only contain *trnI-GAU*, which is different from the species containing large IRs in their CPGs [31]. The boundary of the LSC-IRb is located in the *psbA* gene with 5′-*psbA* located in the LSC and 3′-*psbA* in the IR region. In addition, the PCGs trans-spliced across the boundary of the LSC and IR regions were in the CPGs in some other plants (e.g., *rps12* of *P. edulis* [4] and *Gentiana*) [30], in which the exon1 of *rps12* gene was observed in the LSC region, and the other two exons were in the IR regions. On the other hand, in the *P. massoniana* CPG, the 5′ exon of the *rps12* gene was located in the LSC region, far from the other two remaining exons, which were observed in the SSC region because of the loss of large IRs in the *P. massoniana* CPG (Figure 1) [32].

### 2.3. Structural and Gene Rearrangements

**Figure 2:**
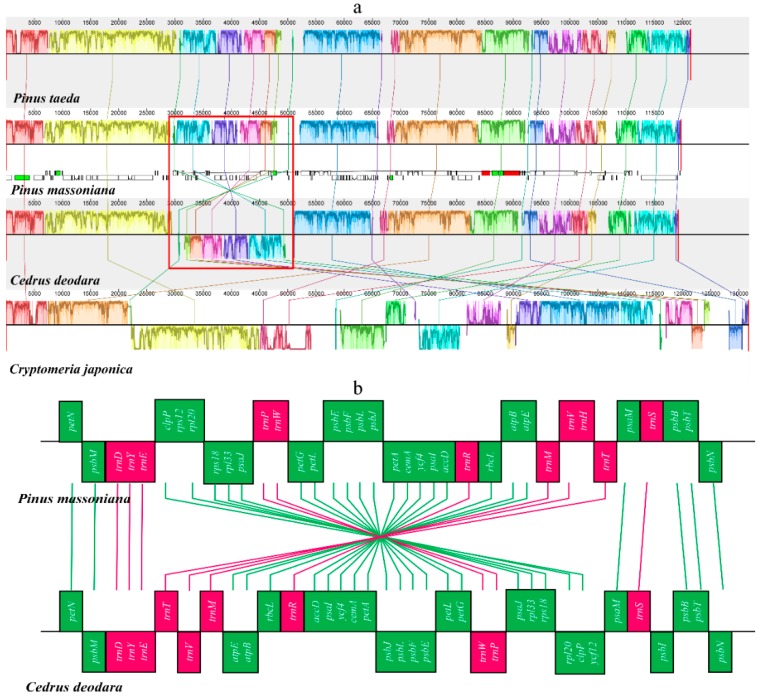
Synteny and rearrangements detected in chloroplast genome sequences of four Coniferous species using the Mauve multiple-genome alignment. (**b**) is a schematic illustration of the red frame part of the (**a**). (**a**) Color bars indicate syntenic blocks, and connecting lines indicate correspondence blocks; (**b**) Green boxes means protein-coding genes; red boxes means tRNAs. Boxes above and below the main line indicate the forward and reverse direction, respectively.

We compared *P. massoniana* organization with other conifers: *P. taeda*, *Cedrus deodara*, and *Cryptomeria japonica*. There were no structural variations between *P. massoniana* and *P. taeda*, while some inversions were observed among *Pinus* (*P. massoniana* and *P. taeda*), *C. deodara*, and *C. japonica* (Figure 2a). Figure 2a shows many structural variations between *P. massoniana* and *C. japonica*, but the former only showed one large inversion of 21,556 bp between the genes *clpP* and *trnT*-GGU in the LSC region based on a comparison with *C. deodara* [4]. The gene order in *P. massoniana* (*clpP*, *rps12*, *rpl20*, *rps18*, *rpl33*, *psaJ*, *trnP*, *trnW*, *petG*, *petL*, *psbE*, *psbF*, *psbL*, *psbJ*, *petA*, *cemA*, *ycf4*, *psaI*, *accD*, *trnR*, *rbcL*, *atpB*, *atpE*, *trnM*, *trnV*, *trnH* and *trnT*) changed to the inverted order in *C. deodara* (*trnT*, *trnV*, *trnM*, *atpE*, *atpB*, *rbcL*, *trnR*, *accD*, *psaI*, *ycf4*, *cemA*, *petA*, *psbJ*, *psbL*, *psbF*, *psbE*, *petL*, *petG*, *trnW*, *trnP*, *psaJ*, *rpl33*, *rps18*, *rpl20* and *clpP*) (Figure 2b). Large IRs play important roles in stabilizing the CPGs against major structural variations, and the loss of large IRs could result in shorter intergenic spacers [6], more gene loss, and structural rearrangements [7,9]. Thus, the loss of the large IRs in the CPGs of *P. massoniana* and *C. deodara* may be the main cause of rearrangements in gene block order. The rearrangement of segments in CPGs are phylogenetically informative [4,6], and may be considered as useful tools in phylogenetic analyses.

### 2.4. Microsatellite Polymorphisms

**Figure 3:**
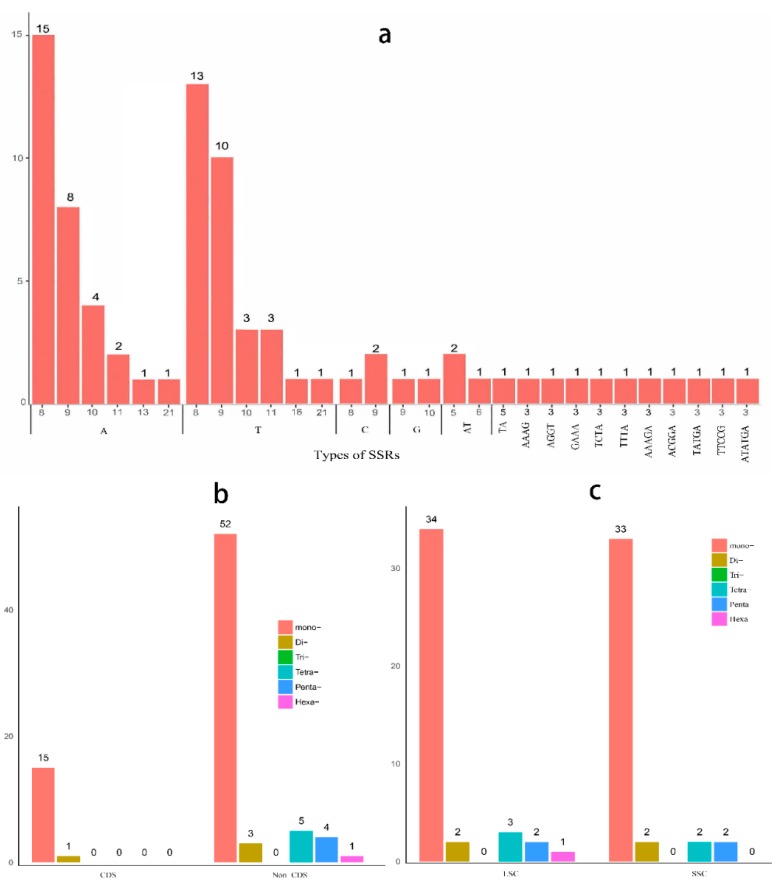
Distribution of each simple sequence repeats (SSR) category in chloroplast genome (CPG) of *Pinus massoniana*. (**a**) Distribution of each SSR category in whole chloroplast genome; (**b**) Distribution of each SSR category in the coding sequence (CDS) and non-CDS of CPG; (**c**) Distribution of each SSR category in LSC and SSC of CPG.

Microsatellites are SSRs with motifs of 1–6 bp that are distributed throughout the nuclear or plasmid genomes. In this study, 81 SSRs were detected in the *P. massoniana* CPG, with minimum SSRs of eight, five, four, three, three, and three for mono-, di-, tri-, tetra-, penta- and hexa-nucleotides, respectively (Figure 3). The majority of the SSRs are mono-; these account for 82.7% of the total SSRs (Figure 3a). Of these, 92.5% have A/T motifs, while only 7.5% have C/G motifs, which is in accordance with the trend of A/T-enrichment in CPGs [11,33]. Sixty-five of the 81 SSRs were found to be located in non-coding sequences (non-CDSs), while 19.75% of the total SSRs were located in the coding sequence (CDS) (Figure 3b). The similar distribution of numbers and types of SSRs were observed in the LSC and SSC regions of the *P. massoniana* CPG (Figure 3c). Among the 10 PCGs containing SSRs, two genes (*ycf1* and *ycf2*) contained two or more SSRs motifs, while the other eight genes (*chlL*, *petA*, *rpl32*, *rpl33*, *rpoC2*, *rps7*, *rps19* and *ycf4*) contained one mono-SSR of the A/T type. Although gene sequences are strongly conserved in CPGs, some SSR variations had already been detected in CDSs [34]. Some differences among numbers and types of SSRs were observed in CPG genes [4,35]. The *clpP* and *ndhA* genes contain tri- and tetra-SSRs, respectively, in the *P. edulis* CPG [4]. Additionally, five genes (*psbC*, *accD*, *cemA*, *petA*, *ycf2* and *ycf1*) were found that contained two or more SSRs in the *Ananas comosus* CPG [35]. These all had different distributions from the SSRs in the *P. massoniana* CPG [35]. In addition, the SSR variations could result in frameshift mutations or loss of gene functions [34]. These are potential causes of gene loss or pseudogene formation.

### 2.5. Loss of ndh Genes

**Figure 4:**
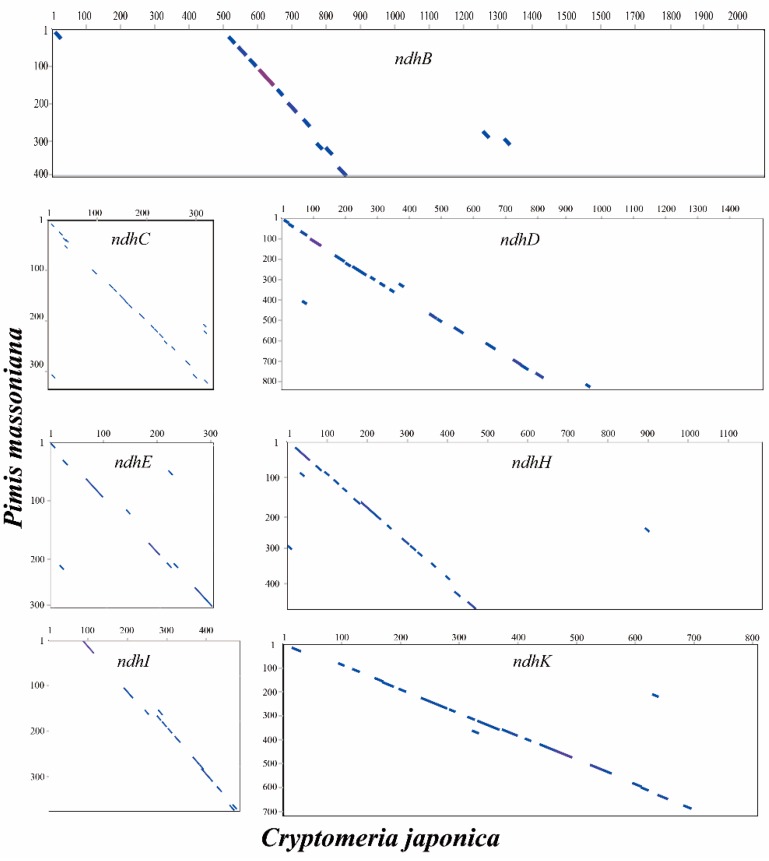
Dotpot analysis of seven *ndh* genes between *P. massoniana* and *Cryptomeria japonica.*

The *ndh* genes are located in nuclear, mitochondrial, and CPGs that can encode the NAD(P)H-dehydrogenase-like (NDH). With the exception of some species in Pinaceae [17], Orchidaceae [15], and Gnetales [36], 11 *ndh* genes, including *ndhA–K*, were found in most land plant CPGs. In this study, the comparisons with CPGs of *C. japonica* [9] and *G. biloba* [3] indicated that the masson pine CPG lacks all 11 intact *ndh* genes. Among these *ndh* genes in the masson pine CPG, the four genes *ndhA*, *ndhF*, *ndhG* and *ndhJ* have been lost completely, the *ndhC* and *ndhE* genes remain as pseudogenes because of short insertions or short deletions, and the other five *ndh* genes (*ndhB*, *ndhH*, *ndhD*, *ndhI* and *ndhK*) remain as truncated pseudogenes (Figure 4). As shown in Figure 4, the *ndhI* gene lost its 5′ end, while the other four truncated pseudogenes (*ndhB*, *ndhH*, *ndhD* and *ndhK*) lost their 3′ ends. *ndhK* lacks a short sequence from the 3′ end (79 bp), but it was not classified as a truncated pseudogene in a previous study of *Pinus thunbergii* [37].

In previous studies, two phenomena of *ndh* gene loss have been observed. Some *ndh* genes are lost completely or transferred to nuclear genomes. For example, some non-functional plastid *ndh* gene fragments are found in the nuclear genome of *Picea abies* [17]. Other *ndh* genes are retained as pseudogenes in CPG which are absent in the nuclear genome. For example, most of the *ndh* genes lost from CPGs are not located in the nuclear genomes of particular orchids [15]. In addition, the chloroplast NDH complex encoded by *ndh* genes is not necessary in some photoautotrophic plants [15,16,17,18]. Thus, we hypothesize that the *ndh* genes (*ndhA*, *ndhF*, *ndhG* and *ndhJ*) that were absent completely in the CPG of *P. massoniana* have been transferred to the nuclear genome. However, this still needs further verification.

### 2.6. Contraction and Expansion of Short IRs in Pinus

Large IRs play vital roles in maintaining the stability of the CPG [6,7], and the loss of a large IR could result in some variations in the gene content and genome structures in CPGs [6,11,12]. IR expansion and contraction exists in many land plants [5]. However, there are no large IR regions in the CPGs of the conifers (e.g., Pinaceae and Taxodiaceae). They have been replaced by short IRs (400–500 bp) [21,25,38], but these do not exist in some conifers [7,9].

**Figure 5:**
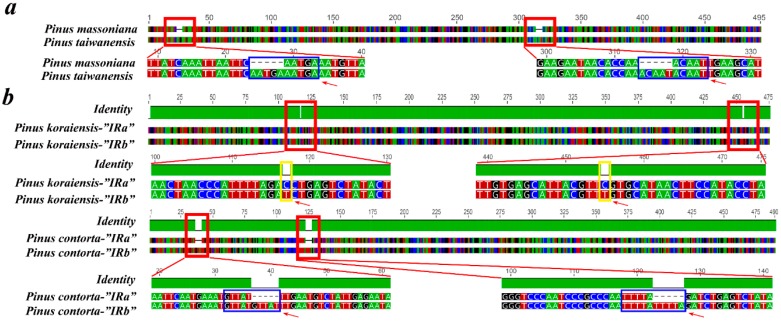
Variations of inverted repeats (IRs) using multiple alignment. (**a**) Variations between *P. massoniana* and *P. taiwanensis*; (**b**) variations between “IRa” and “IRb” in *P. koraiensis* and *P. contorta*. Variations are in red frames: Single Nucleotide Polymorphisms (SNPs) are in yellow frames; microsatellites are in blue frames.

In the present study, we observed a pair of short IRs (485 bp) and an absence of large IRs. However, some variations in size and sequence were also found in short IRs among species in *Pinus*. The alignment results of “IRa” and “IRb” (screened using the reference sequences from *P. taiwanensis*) showed that the main causes of the variations are as follows: (1) Insertion or deletion of short sequences (variations in repeat numbers of microsatellite motifs). The IRs of *P. massoniana* are 10 bp shorter than those of *P. taiwanensis* because of the loss of a 5-bp microsatellite motif (AATGA and ACAAT) in two loci of IRs of *P. massoniana* (Figure 5a); (2) Non-synchronized variations between “IRa” and “IRb” (e.g., single nucleotide polymorphisms and SSRs). The alignment indicated that two single nucleotide polymorphisms and the loss of two 5-bp microsatellite motifs (GTTAT and TTTTA) were found in the “IRa” regions of *Pinus koraiensis* and *Pinus contorta*, respectively (Figure 5b). These could lead to variations in the size and sequence of short IRs in conifer CPGs, but they are not the main causes of large IR expansion and contraction. Large IRs can provide enhanced copy-correction activity because the rates of nucleotide substitution in IRs are several times slower than in SSC regions [5,8]. In addition, the loss of gene copies in large IRs could result in IR contraction, similar to how the loss of an *ycf2* copy led to IR contraction in *G. biloba* [3]. Although the loss of large IRs has been confirmed in species of Cephalotaxaceae [12], Taxodiaceae [10,11] and Legumes [8], determining the main cause requires further study.

### 2.7. Phylogenetic Analysis

CPG sequences have been widely used in the phylogenetic analyses of land plants [5,12,26]. In particular, some CDSs of *matK*, *rbcL* and *rpoB* genes and non-CDSs of *atpF-atpH*, *trnH-psbA* and *psbK-psbI* have been used in phylogenetic studies [19,20]. However, few CDSs and non-CDSs in CPGs are not informative enough when studying closely related species and cultivars. Thus, whole CPG sequences that contain more variation could play important roles in phylogenetic analyses.

**Figure 6:**
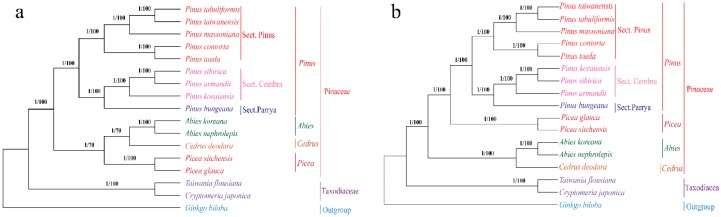
Phylogenetic tree constructed by maximum likelihood (ML) and Bayesian inference (BI) methods based on whole chloroplast genome sequences and 56 shared protein-coding genes of 16 conifers. (**a**) Phylogenetic tree based on whole chloroplast genome sequences of 16 conifers; (**b**) Phylogenetic tree based on 56 shared protein-coding genes of 16 conifers chloroplast genomes; *Ginkgo biloba* as an outgroup; BI posterior probability/ML bootstrap support values were listed at each node.

In the present study, phylogenetic analyses were performed based on the whole CPG sequences of 16 conifers, using *G. biloba* as an outgroup. We obtained a congruent phylogenetic tree with different support values using maximum likelihood (ML) and Bayesian inference (BI) approaches (Figure 6). All 16 conifers could be discriminated completely into two clades of Pinaceae and Taxodiaceae with high BI posterior probability and ML bootstrap support. Nine pine species were divided into two groups (sect. Pinus and sect. Cembra + sect. Parrya) (posterior probability = 1 and ML bootstrap support = 100), which supports the results of Parks et al [26]. In addition, *P. massoniana* is closely related to *P. taiwanensis* and *P. tabuliformis*, but not to *P. taeda* or *P. contorta*. These results corroborate previous studies [7,12,21]. However, the phylogenetic relationships based on whole CPG sequences between *Pinus*, *Abies*, *Cedrus*, and *Picea* in this study conflicted with previous studies. Here, *Pinus* was placed into a clade, while *Abies*, *Cedrus*, and *Picea* were placed into another clade, which is consistent with conventional plant taxonomy (Figure 6). In some previous studies, *Pinus* and *Picea* were placed into a clade, and *Abies* and *Cedrus* were placed into another clade, which is similar to our phylogenetic analyses based on 56 shared PCGs of 17 species (Supplementary Table S2). However, it is worth noting that the phylogenetic trees in previous studies were constructed based on shared PCGs [7,21] and low and high heterotachous data sets [12], while whole CPG sequences were used in our phylogenetic analyses. Thus, differences may be observed among the phylogenetic relationships established based on different CPG regions. As we all know, the chloroplast genome sequences are conserved and mainly embodied in the conservation of PCGs sequences. The result of average similarity scores of 56 shared PCGs sequences show that the similarity scores of shared PCGs sequences (average similarity score = 0.9592) in Pinaceae is much greater than that of Pinaceae-Ginkgoaceae (average similarity score = 0.8616) (Supplementary Table S3). Hence, particular CPG regions (like PCGs) that contain few variations among species are not suitable for establishing phylogenetic relationships of closely related species in *Pinus*, while whole CPG sequences may be powerful tools in phylogenetic analyses of closely related species or cultivars.

## 3. Materials and Methods

### 3.1. DNA Source, Template Amplification

The needles of *P. massoniana* were collected from Longyan (Fujiang, China, N 25.25°, E 117.54°). Total DNA was extracted using the Plant Genprep DNA Kit (ZomanBio Inc., Beijing, China) and quantified using a NanoDrop 2000c (ThermoFisher Scientific, Wilmington, DE, USA). The entire CPG was amplified using PCR with 35 primer pairs [22]. Amplicons for these regions averaged ~3.6 kb, and fragments were obtained with PrimeSTAR Max DNA Polymerase (Takara Bio Inc., Dalian, China).

### 3.2. Sequencing, Assembly, PCR-Based Gap Filling and Annotation

PCR products were evaluated using electrophoresis on agarose gels and purified using a Gel Mini purification Kit (ZomanBio Inc., Beijing, China). All of the purified DNA products were sequenced using an ABI 3730 DNA sequencer. Sequence assemblies were performed using CAP3 [39] and manually confirmed against the reference CPG of *Pinus taeda* (KC427273.1).

The genome annotation was performed using a Dual Organellar GenoMe Annotator with default parameters [40], coupled with manual corrections of the start and stop codon positions. All of the tRNAs were identified using tRNAscan-SE v2.0 program (http://lowelab.ucsc.edu/tRNAscan-SE/) [41]. The boundaries of the exons and introns were verified using the BLASTn algorithm (2.6.0, National Center for Biotechnology Information, Bethesda, MD, USA, 2017) against other closely related pine species. The annotation map of the CPG was generated using Organellar Genome DRAW v1.2 program (http://ogdraw.mpimp-golm.mpg.de/) [42].

### 3.3. Simple Sequence Repeat Analysis

The microsatellite motifs were identified using the MIcroSAtellite identification tool (http://pgrc.ipk-gatersleben.de/misa/misa.html) with the following parameters [43]: the minimum repeats of SSRs were eight, five, four, three, three, and three mononucleotides (mono-), dinucleotides (di-), trinucleotides (tri-), tetranucleotides (tetra-), penta-nucleotides (penta-) and hexa-nucleotides, respectively.

### 3.4. Sequence Analysis

To highlight the differences in IRs between some species in Pinaceae and Taxodiaceae, a comparative analysis were conducted by aligning IR sequences using multiple sequence alignment software (MAFFT) [44]. Special attention was paid to the variations among the IR sequences. In addition, the loss of *ndh* genes was found through sequences alignments using MAFFT [44] based on the CPG annotation of *P. massoniana*. Whole-genome alignments were conducted to locate structural differences using MAUVE [45].

### 3.5. Phylogenetic Analysis

The whole CPG sequences of 16 conifers were used in a phylogenetic analysis with *Ginkgo biloba* as the outgroup (Supplementary Table S4). The phylogenetic analysis was carried out based on complete CPG sequences and 56 shared PCGs (Supplementary Table S2). The multiple alignments of the sequences mentioned above were performed on MAFFT [44]. The programs JModeltest2 [46] and Modelgenerator [47] were used to find an optimal substitution model for the subset by taking the Akaike Information Criterion (AIC) values into account [48]. The maximum likelihood (ML) tree of whole CPG sequences (Model: GTR+G) was conducted using RAxML 8.2.7 [49] with a bootstrap of 1000 replicates. A Bayesian inference (BI) analysis was run in MrBayes 3.2.6 with the setting of 1,000,000 generations with trees sampled every 1000 generations [50,51]. The first 25% of trees were discarded as burn-in to estimate the values of posterior probabilities.

## Figures and Tables

**Table 1:** Gene contents of *P.massoniana* chloroplast genome based on genome annotation.

Category	Gene Contents
Subunits of photosystem I	*psaA*, *psaB*, *psaC*, *psaI*, *psaJ*, *psaM^a^*
Subunits of photosystem II	*psbA*, *psbB*, *psbC*, *psbD*, *psbE*, *psbF*, *psbH*, *psbI*, *psbJ*, *psbK*, *psbL*, *psbM*, *psbN*, *psbT*, *psbZ*
Small subunit of ribosome	*rps2*, *rps3*, *rps4*, *rps7*, *rps8*, *rps11*, *rps12^b^*, *rps14*, *rps15*, *rps18*, *rps19*
Large subunit of ribosome	*rpl2^b^*, *rpl14*, *rpl16^b^*, *rpl20*, *rpl22*, *rpl23*, *rpl32*, *rpl33*, *rpl36*
Subunits of cytochrome b/f complex	*petA*, *petB^b^*, *petD^b^*, *petG*, *petL*, *petN*
Subunits of ATP synthase	*atpA*, *atpB*, *atpE*, *atpF^b^*, *atpH*, *atpI*
DNA-dependent RNA polymerase	*rpoA*, *rpoB*, *rpoC1^b^*, *rpoC2*
ChlorophyII biosynthesis	*chlB*, *chlL*, *chlN*
Protease	*clpP*
Maturase	*matK*
Envelope membrane protein	*cemA*
Translation initiation factor	*infA*
Cytochrome c biogenesis	*ccsA*
Subunit Acetyl-CoA-Carboxylate	*accD*
Subunit of rubisco	*rbcL*
Ribosomal RNAs	*rrn4.5*, *rrn5*, *rrn16*, *rrn23*
Conserved open reading frames	*ycf1*, *ycf2*, *ycf3^b^*, *ycf4*, *ycf12*, *ycf68*
Transfer RNA	*trnA-UGC^b^*, *trnC-GCA*, *trnD-GUC*, *trnE-UUC*, *trnF-GAA*, *trnfM-CAU*, *trnG-UCC*, *trnG-GCC^b^*, *trnH-GUG^a^*, *trnI-GAU^ab^*, *trnK-UUU^b^*
	*trnL-CAA*, *trnL-UAA^b^*, *trnL-UAG*, *trnM-CAU*, *trnN-GUU*, *trnP-GGG*, *trnP-UGG*, *trnQ-UUG*, *trnR-ACG*, *trnR-CCG*, *trnR-UCU*
	*trnS-GCU^a^*, *trnS-GGA*, *trnS-UGA*, *trnT-GGU^a^*, *trnT-UGU*, *trnV-GAC*, *trnV-UAC^b^*, *trnW-CCA*, *trnY-GUA*

^a^ Gene-copies in genome; ^b^ Intro-containing gene.

## Supplementary Materials

Supplementary materials are available online.
